# Large Coccinellidae Collected in the Nebraska Panhandle Reveal High Diversity, Rare Species, and Undocumented Plant Associations

**DOI:** 10.3390/insects17101018

**Published:** 2026-10-02

**Authors:** Mathew L. Brust, Louis S. Hesler, William Wyatt Hoback

**Affiliations:** 1 Department of Biology, Chadron State College, Chadron, NE 69337, USA; 2 Hesler Entomological Research & Consulting, Jackson, MS 39211, USA; hexapodsci@gmail.com; 3 Department of Entomology and Plant Pathology, Oklahoma State University, Stillwater, OK 74078, USA; whoback@okstate.edu

**Keywords:** biological control, predator, occurrence records, sampling methods, polyphagy, ladybug, declining, invasive

## Abstract

Museum records and recent field surveys provide new insights into the diversity, collection methods, seasonal ecology, and plant associations of lady beetles (Coleoptera: Coccinellidae) in western Nebraska. More than 2500 specimens exist, with data on collection methods for 1882 specimens representing museum holdings and surveys conducted between 2012 and 2025. Most specimens were obtained by hand collection (1244; 66%), followed by sweep net sampling (292; 15%) and blacklight traps (261; 13.8%), whereas yellow pan traps produced 85 (4.5%) of all specimens. These proportions represent the composition of the collection and should not be interpreted as estimates of relative sampling efficiency because effort was not standardized among methods. More than half of all museum specimens of *Adalia bipunctata*, *Harmonia axyridis*, *Hippodamia tredecimpunctata tibialis* Timberlake, and *Myzia interrupta* (Casey) originated from a single continuously operated blacklight trap, while 26% of *Coccinella transversoguttata* were captured in yellow pan traps. Plant associations were documented for 1070 specimens and revealed recurring species-specific patterns in available records. Notably, 50% of *Coleomegilla maculata lengi* were collected on knotweeds (*Polygonum* spp.), 27% of *Hippodamia parenthesis* were associated with alfalfa, and two *Scymnus* species were collected from elm. Native sunflowers (*Helianthus* spp.) were particularly important, with over 75% of *Coccinella monticola*, *C. novemnotata*, and *Hippodamia convergens* collected apparently feeding on this host. Observations of lady beetles associated with sunflowers, in the absence of aphids or other apparent prey, suggest late-season phytophagy or exploitation of plant exudates rather than predation by adult beetles. Focused surveys with broadly similar search effort revealed striking interannual variation in detections, with several species frequently encountered in 2024 detected infrequently or not detected in 2025. Collectively, these records document the persistence of rare native coccinellids in western Nebraska, identify potentially important plant associations and detection methods, and provide baseline occurrence and natural-history information for future standardized monitoring.

## Simple Summary

Although several native North American lady beetle species appear to have vanished from large portions of their former range, some remain present in the Nebraska Panhandle, though mostly in relatively low numbers. The entomology collection at Chadron State in northwestern Nebraska contains numerous lady beetle specimens, with a significant portion collected in the past 25 years. In many cases, collecting methods were included on the collection labels. These records provide information on species occurrence, historical and recent distributions, plant associations, and the collection methods by which particular species were detected. Because sampling effort and methods varied among locations and years, specimen numbers cannot be interpreted directly as estimates of population abundance or as comparisons of sampling-method effectiveness. We report on the occurrence of rare ladybeetle species and highlight potential late-season phytophagy and identify collection approaches that may complement conventional surveys and potentially increase detection of some species. These observations provide a foundation for future standardized monitoring of lady beetle communities in the Great Plains.

## 1. Introduction

Insects are the most diverse group of multicellular organisms with more than a million described species and an estimated five to ten million extant species worldwide [1,2,3]. They occupy virtually every terrestrial and freshwater ecosystem and play critical roles in regulating plant populations as herbivores, seed dispersers, pollinators, and decomposers. Additionally, insects are predators, parasitoids, and parasites that regulate vertebrate and invertebrate populations [4,5]. Through these ecological interactions, insects affect food-web dynamics, influence primary productivity, recycle nutrients, and provide ecosystem services valued at hundreds of billions of dollars annually [6,7]. However, accumulating evidence over the past three decades suggests that many insect populations are declining in abundance, biomass, and geographic distribution [8,9,10,11], and many species are believed to be at risk of extinction.

Although the decline or disappearance of insects has been reported for decades [11], quantitative evidence has become increasingly available through long-term monitoring programs, standardized trapping efforts, examination of museum collections, and meta-analyses. Following the report of a 75% decline in flying insect biomass over a 27-year period in protected areas throughout Germany, widespread research has been conducted [8]. Similar reductions in insect biomass and abundance have been reported from portions of Europe, North America, Asia, and South America, although the magnitude of change varies considerably among taxa, habitats, and geographic regions [10,12,13].

Initial interpretations of insect decline emphasized catastrophic losses across entire insect communities, leading to the term “insect apocalypse”. However, subsequent research has demonstrated that global insect trends are considerably more complex than originally portrayed. Comprehensive reviews and meta-analyses indicate that while numerous insect groups exhibit substantial declines, others remain relatively stable or even increase locally in response to land-use change, climate change, pesticide use, urbanization, or the effects of invasive species [10,13]. Consequently, current scientific consensus recognizes that insect declines are taxon-specific, geographically heterogeneous, and strongly influenced by local environmental and land-use conditions rather than representing a uniform phenomenon [11,14].

Greater understanding of potential insect declines has emerged largely through improvements in examination of long-term datasets. Recently, Crossley et al. [13] synthesized more than five thousand insect time series across North America and reported relatively stable overall abundance; however, declines among some taxonomic groups and functional guilds were observed. Wagner et al. [11] noted that variation among study results often reflects differences in sampling methodology, habitat type, geographic coverage, and taxonomic focus. Additionally, the absence of taxa catalogues of species, few recognized terrestrial bioindicator taxa, and limited long-term data sets complicates interpretation. Research that examines taxa trends, population changes, and threats is critically needed, especially on groups that can be easily identified and monitored by the public through citizen science platforms over large areas. For example, examination of trends for the well-studied butterflies using large data sets collected over wide geographic areas revealed that total abundance of North American butterflies has declined by 22% from 2000 to 2020 [15]. However, even for such a popular group of insects, caution is needed when relying on public data as abundance and seasonal patterns may be difficult to quantify using citizen science alone [16].

Lady beetles (Coleoptera: Coccinellidae), especially those with adults > 4 mm, represent a potential model group for biodiversity inventories and public monitoring [17,18]. The family has more than 6500 described species distributed among approximately 360 genera and occurs worldwide in most terrestrial habitats except for polar extremes and the highest alpine environments [19]. Lady beetles are among the most recognizable and ecologically important groups of predatory insects worldwide [18]. Although commonly recognized by their aposematic coloration and patterned elytra, coccinellids exhibit remarkable ecological diversity and include predatory, phytophagous, fungivorous, and pollen-feeding genera. In temperate North America, most are predaceous on herbivorous arthropods, especially aphids, and most species encountered are important natural enemies of agricultural and forest pests [20]. The larger species are generally easily identified and are well-represented in historical museum collections.

The bright colors and predaceous habits of many Coccinellidae make them, except for the multicolored Asian lady beetle, *Harmonia axyridis* (Pallas), popular with members of the public [21,22]. Many species occupy both agricultural and natural ecosystems, respond rapidly to environmental change, and provide economically valuable biological control services through predation on herbivorous arthropods.

Unlike many insect groups experiencing poorly documented declines, evidence for changes within North American lady beetle communities is unusually robust because of the availability of museum collections spanning more than a century. Historical specimens deposited in natural history collections have allowed researchers to reconstruct temporal changes in species distributions, morphometrics, abundance, and community composition across broad geographic scales [17,23,24]. These collections have revealed that several formerly widespread native species have undergone dramatic declines since the latter half of the twentieth century.

Among the most notable examples is the ninespotted lady beetle, *Coccinella novemnotata* Herbst. Historically one of the most common aphid predators across the northern United States and southern Canada, this species experienced rapid declines beginning in the 1980s and is now rarely encountered across much of its former range [25,26]. Similar population reductions have been documented for *Coccinella transversoguttata richardsoni* Brown, *Adalia bipunctata* (Linnaeus), and several *Hippodamia* species, including regional declines of *Hippodamia convergens*, although trends for these species vary geographically [27,28,29,30,31,32,33]. The apparent disappearance of formerly abundant lady beetles has generated considerable concern because these species historically contributed substantially to aphid suppression in both agricultural and natural ecosystems.

The causes of native lady beetle declines remain an active area of investigation and likely involve multiple factors. Habitat loss associated with agricultural intensification has reduced the availability of overwintering sites, flowering resources, and semi-natural habitats that support native populations throughout the year [34]. Increased pesticide use, particularly broad-spectrum insecticides, may directly reduce survival while simultaneously altering prey availability and disrupting predator communities [35]. Climate change likely has affected developmental rates, voltinism, phenology, and overwintering survival, potentially influencing competitive interactions among native and introduced species [36].

Perhaps the most widely discussed driver of community restructuring has been the establishment of non-native coccinellids introduced for biological control. The seven-spotted lady beetle, *Coccinella septempunctata*, introduced from Europe during the twentieth century, rapidly expanded throughout North America following its establishment in the 1970s [37,38]. *Harmonia axyridis* became widely established during the 1990s and now occupies nearly every contiguous U.S. state and much of southern Canada [39]. Both species are highly polyphagous, exhibit strong dispersal ability, and frequently attain high population densities in both agricultural and urban habitats.

Nebraska is a state within the USA located in the middle of the Great Plains of North America. The western third of Nebraska is typified by dominant shortgrass prairie interspersed with pine and riparian woodlands [40]. Much of the prairie habitat has been utilized for ranching or converted to irrigated cropland [41].

Western Nebraska remains an understudied area for lady beetles. However, two recent surveys of lady beetles in western Nebraska have documented the persistence of increasingly rare lady beetle species such as *C. novemnotata* and *C. transversoguttata richardsoni* along with introduced species such as *C. septempunctata* [42,43]. Nonetheless, both papers noted the need for additional research in western Nebraska to determine the resource use by native lady beetles and mechanisms of their co-existence with non-native lady beetles.

Various methods may be used to study native lady beetles in western Nebraska, including natural history observations and an assessment of lady beetles within regional natural history collections [42,44]. Beginning in 2010, the first author observed rare lady beetles near the small city of Chadron, Dawes County, western Nebraska, and the surrounding natural areas. Chadron is home to Chadron State College (CSC), a small four-year school that maintains an insect collection with specimens dating from the 1930s to the present; however, a comprehensive inventory of the lady beetle diversity in the CSC museum collection has not been conducted.

Because the CSC collection accumulated through multiple collection methods and levels of sampling effort, we use these specimens primarily as occurrence and natural-history records rather than as a standardized historical measure of relative abundance. The objectives of this study were to document large lady beetle diversity in the CSC museum collection and add recent observations. During field surveys, new aspects of lady beetle biology were observed, including phytophagy by many predominantly predatory species. These observations, along with biological notes about rare lady beetle species, are likely to improve field surveys and provide guidance for characterizing lady beetle community changes elsewhere. Appendix A lists specific details about each species present in the CSC collection.

## 2. Materials and Methods

**Figure 1:**
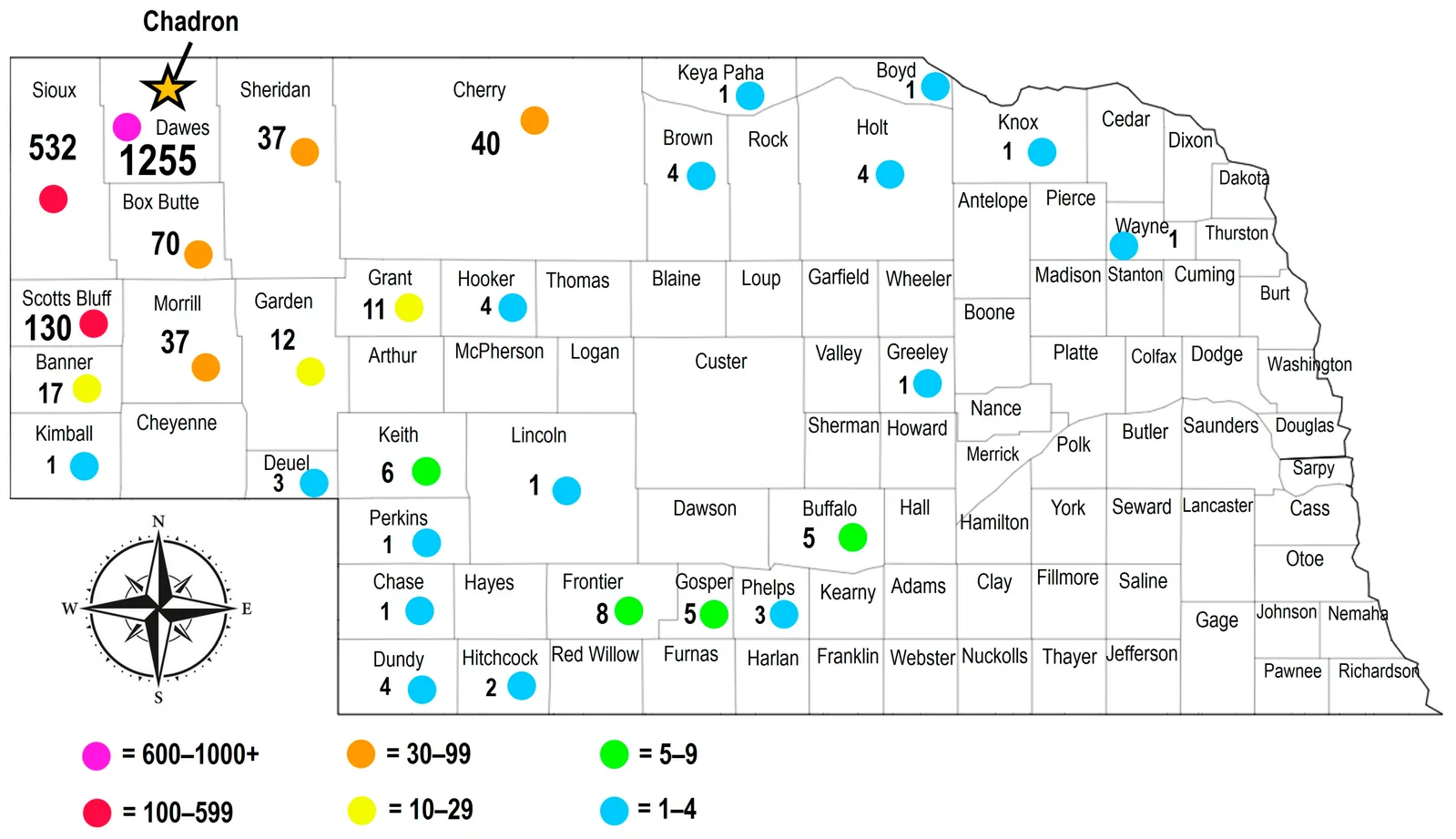
Number of total Coccinellid specimens in CSC collection based on county of collection.

For each specimen in the CSC coccinellid collection, species was determined, and label information was transcribed to a database. This information included state or province, county, location, and, if noted, date and collector name. When present, collection method and associated plant were also recorded. Most adult lady beetles have been collected within 300 km of Chadron, with >50% of the specimens being collected in Dawes County (Figure 1).

Specimens were identified using a variety of resources, particularly [45]). Five specimens are listed as *Hippodamia apicalis/expurgata* because of the high degree of morphological similarity of these species. However, it is likely that all 5 CSC specimens collected in Nebraska are *Hippodamia expurgata* Casey, as *Hippodamia apicalis* Casey is not expected to occur in Nebraska [45]. The first author performed identifications based on morphology, with confirmations made by the second author. All specimens, including ambiguous morphospecies (e.g., *Hippodamia apicalis/expurgata* and putative *Hippodamia quindecimmaculata* Mulsant), were diagnosed only by external morphology. These specimens and all voucher specimens are securely deposited and accessible within the CSC insect collection for future taxonomic diagnosis.

The CSC collection represents specimens accumulated over many decades by different collectors using different methods and levels of sampling effort. Consequently, museum specimen totals were treated as occurrence and collection records rather than standardized estimates of field abundance. Percentages associated with collection methods or host plants describe the representation of specimens within the available dataset and were not used to calculate capture efficiency, detection probability, or relative population abundance.

Similarly, absence of a species from the collection during a particular year or period was not interpreted as evidence of true absence from western Nebraska. Such gaps may reflect biological rarity, variation in collecting effort, changes in collection methods, differences among collectors, or incomplete detection.

Lead author ML Brust joined the faculty at Chadron State College in 2006 and has assessed insect diversity and increased the museum holdings by collecting vouchers of various insects, including lady beetles. Collections consisted of examination of bycatch from sweep net samples in alfalfa and elm (*Ulmus* sp.) and from USDA grasshopper sweep samples of rangelands from multiple locations across the western half of Nebraska. Standardized sweeps (38 cm-diameter net, 20 sweeps per sample, one “figure-eight” per sweep, using low and fast sweeps) of rangeland plants were conducted, and the contents of the sweep net were bagged and frozen. Over the past 15 years, approximately 150 bagged samples have been examined each year.

Although this component of the sampling used a standardized sweep protocol, the total museum dataset also incorporates specimens obtained by hand collecting, blacklight trapping, yellow pan trapping, and other collection activities that were not standardized to equivalent effort. Therefore, comparisons among collection methods are descriptive and represent the proportion of archived specimens obtained by each method rather than formal tests of method effectiveness.

In 2024 and 2025, ML Brust conducted focused visual searches for Coccinellidae. Each year, 40–50 h were spent searching plants on roadsides for lady beetles across 15–25 days. Many of the same locations were visited at the same time of year in both years. Focal plants included knotweed (*Polygonum* spp.), mugwort (*Artemesia vulgaris*), elm, and sunflower (*Helianthus* sp.), which were examined visually, and beetles were hand-collected. In most cases, the plants were only identified to genus. For example, many specimens collected from native sunflowers along roadsides were recorded as *Helianthus* sp. For these, the plant was either *Helianthus annuus* L. or *Helianthus petiolaris* Nutt., which typically co-occur in many areas across western Nebraska.

Because search effort was broadly similar between 2024 and 2025, numbers observed or collected during these targeted surveys provide evidence of interannual variation in detections. However, these surveys were not designed as a replicated population-monitoring program, and differences between years are therefore not interpreted as quantitative estimates of changes in population density.

Throughout the manuscript, historical museum records and recent survey data are therefore distinguished conceptually. Museum records are used principally to document occurrence, collection history, phenology, and plant associations, whereas the 2024–2025 observations provide a more focused comparison of recent detections under broadly similar search effort.

## 3. Results

Chadron State College (CSC) houses an insect collection with over 2500 lady beetle specimens. Of these, about 2200 were collected in Nebraska, while the remainder were collected in other states, especially Wyoming, South Dakota, and Wisconsin. For this study, only specimens recorded from Nebraska were considered. Approximately 87% of all the Nebraska coccinellid specimens in the collection were collected in Dawes, Scotts Bluff, or Sioux Counties (Figure 1). Therefore, coccinellids from western Nebraska constitute the majority of lady beetle specimens in the CSC collection.

We found 35 identified species of lady beetle (Table 1). The most-frequently represented species were the native *H. convergens* and introduced *C. septempunctata*, which together accounted for slightly more than 50% of all specimens. Six species were represented as singletons and 22 species represented less than 1% of specimens in the collection. Because collection effort varied, representation in the collection should not be interpreted as a standardized estimate of relative field abundance.

### 3.1. Collecting Methods

Collection information exists for 1882 of the 2200 Nebraska lady beetle specimens from the museum and recent collections. Specimens were most commonly obtained by hand collection (1244 specimens: 66% of the total), while light traps and sweep net samples generated 261 (14%) and 292 (15%) specimens, respectively. Yellow pan traps accounted for the smallest proportion of specimens with recorded collection methods, with 85 (4.5%) specimens collected. These percentages describe the representation of collection methods among archived specimens and are not measures of sampling efficiency because effort differed substantially among methods.

Of note, a substantial proportion of the CSC specimens of several species originated from a single continuously operated blacklight trap. More than half of all the specimens in the museum collection of *Adalia bipunctata*, *Harmonia axyridis*, *Hippodamia tredecimpunctata. tibialis*, *Myzia interrupta*, and *Olla v-nigrum* (Mulsant) were collected at this light trap. Also noteworthy was that 26% of *C. transversoguttata richardsoni* were collected in yellow pan traps (Table 2). These records identify potentially useful species-specific detection patterns but do not demonstrate that blacklight or pan trapping was more effective than other collection methods.

### 3.2. Plant Associations Recorded for Hand- and Sweep-Collected Specimens

Plant associations were noted for 1070 (86%) of the specimens that were collected by sweepnetting or by hand. Of these, 115 were obtained from sweep samples of alfalfa, clover, knotweed, and elm, while the vast majority of lady beetles were hand-collected during searches of plants (Table 2).

Despite extensive sweep sampling associated with grasshopper surveys in rangelands and additional sweeping of alfalfa and elm, relatively few coccinellid specimens occurred as bycatch. Because total effort differed between sweep sampling and targeted visual searches, these numbers cannot be used to compare the effectiveness of the two methods. However, approximately 27% of specimens of *Hippodamia parenthesis* Say. were associated with alfalfa (*Medicago sativa*). All specimens of *Scymnus brullei,* one of two *S. caudalis* with available records, were obtained from sweep samples of elm (*Ulmus* sp.) leaves, most likely all among American elm (*Ulmus americana*). Because *Scymnus* and other small-bodied coccinellids are likely to be under-detected by visual searches, these observations are particularly useful for identifying methods and vegetation that could be incorporated into future standardized surveys.

Among hand-collected specimens, 50% of *Coleomegilla maculata* (De Geer) were collected from knotweeds (*Polygonum* sp.). Many lady beetle specimens with plant information were recorded from native, non-cultivated sunflowers (*Helianthus* spp.). More than 20% of the specimens of *Anatis mali* (Say), *Coccinella septempunctata*, *Hippodamia expurgata*, and the putative *Hippodamia quindecimmaculata* were collected from *Helianthus* species. More than 75% of all specimens of *Coccinella monticola* Mulsant, *C. novemnotata*, and *H. convergens* were collected on *H. annuus* or *H. petiolaris*. Of the *Anatis mali* collected on *Helianthus* species, one was collected on *H. annuus* or *H. petiolaris,* while the remainder were collected on *H. nuttallii*. These percentages describe specimen-level plant records and are not estimates of plant preference or habitat selection.

**Figure 2:**
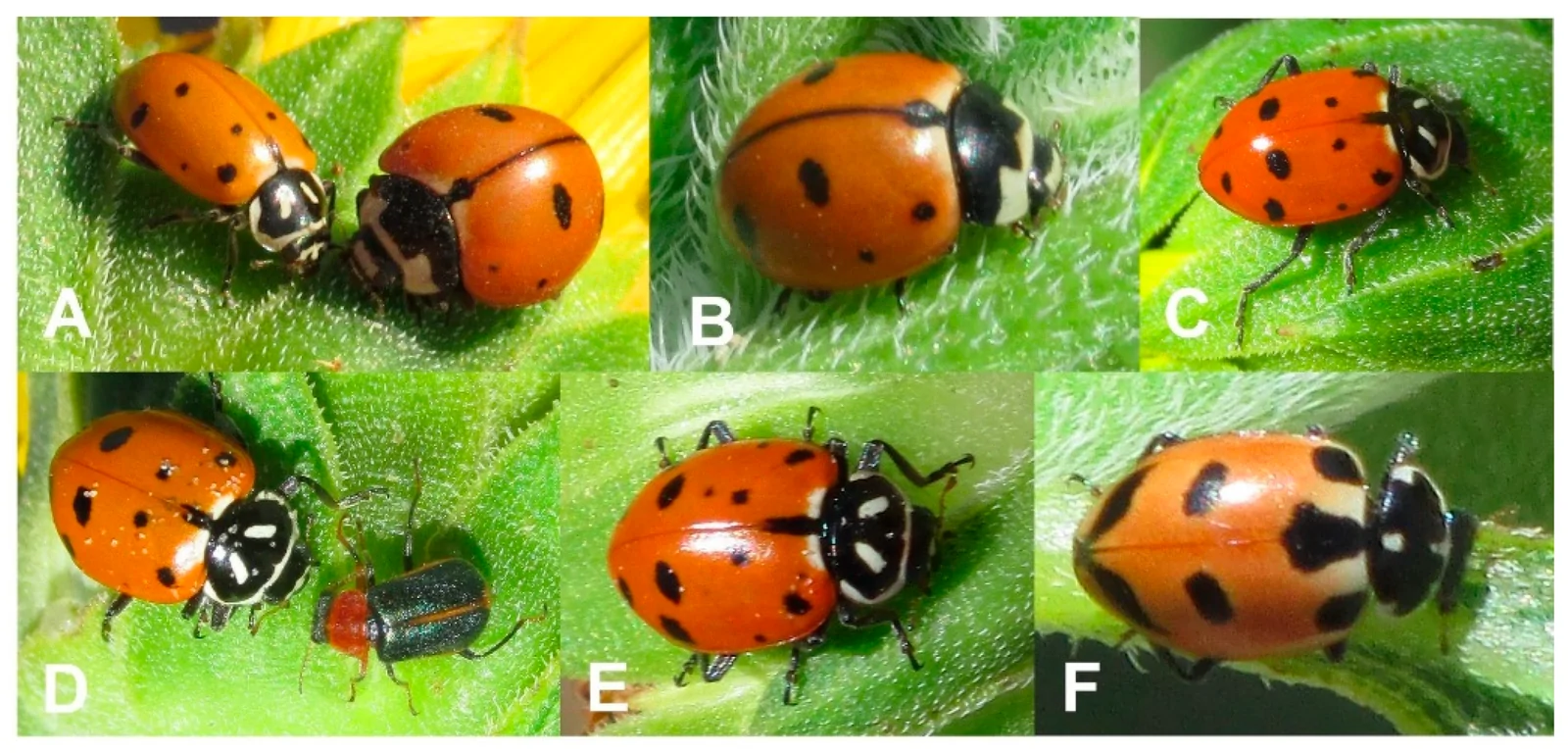
Examples of Coccinellidae in western Nebraska, USA, apparently feeding on sunflower (*Helianthus* sp.) sap: (**A**) *Coccinella novemnotata* and *Hippodamia convergens*, Dawes County, about 14.5 km SSE of Chadron, along Deadhorse Road, 3 July 2024; (**B**) = *Coccinella novemnotata*, Dawes County, about 14.5 km SSE of Chadron, along Deadhorse Road, 13 July 2024; (**C**,**D**) = *Hippodamia convergens*, County, 2.7 km NNE of Harrison, along Sowbelly Road, 31 August 2024; (**E**) = *Hippodamia convergens*, Sioux County, 4.3 km NNE of Harrison, along Sowbelly Road, 1 September 2024; (**F**) = *Hippodamia expurgata*, Sioux County, 2.7 km NNE of Harrison, along Sowbelly Road, 1 September 2024.

**Figure 3:**
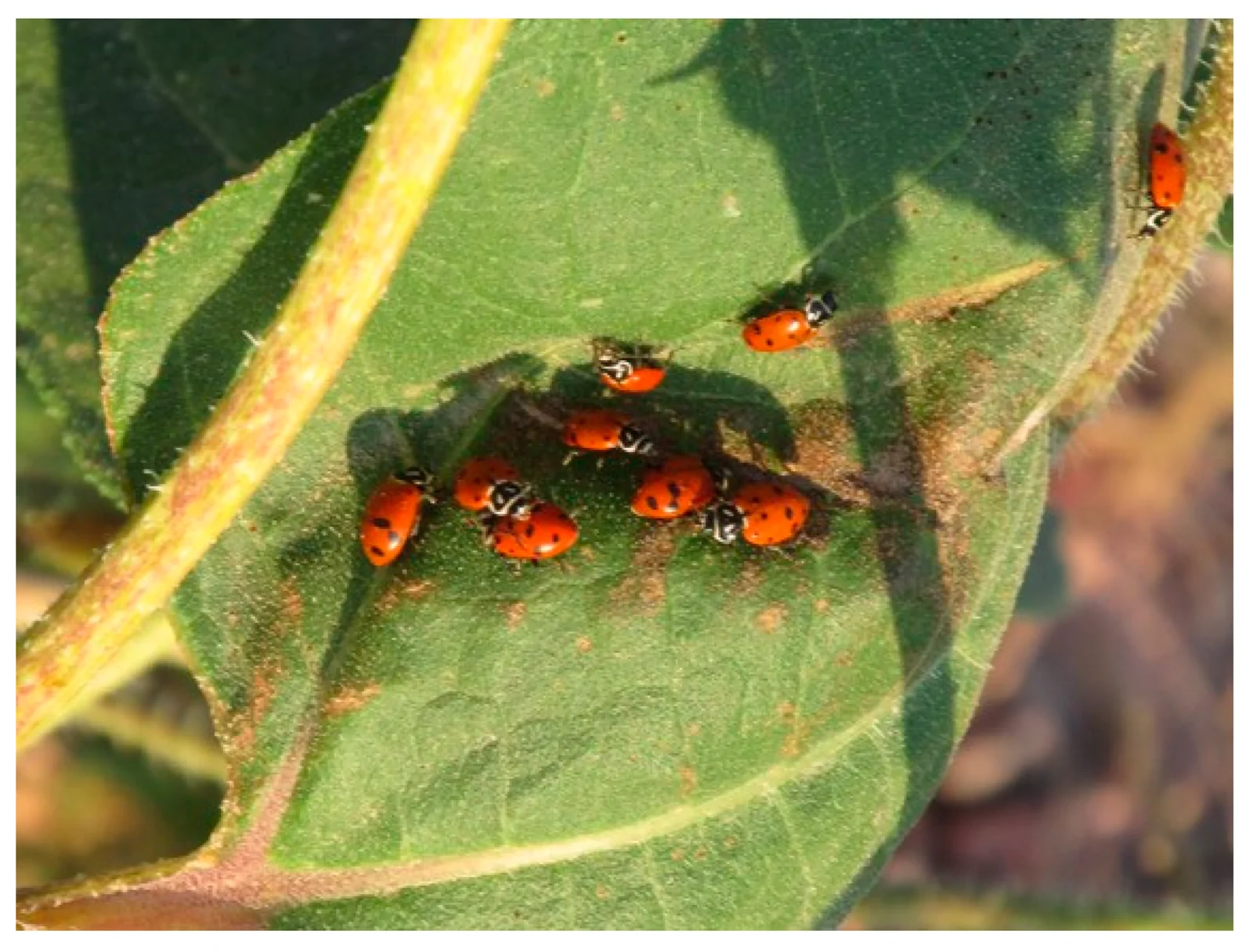
Example of Coccinellidae likely feeding on sunflower leaf material in a group about 8.8 km S of Chadron, Nebraska, USA, along King Canyon Road, on *Helianthus* sp., 7 September 2024. In this case, the leaf was curled over, and the beetles were hidden within the curled leaf. Note the extensive leaf damage. Beetles were a mix of *Hippodamia convergens* and *H. glacialis*.

Although specific behavior on host plants was not recorded on labels, observations by the first author have revealed previously undocumented host plant-associated behavior. Among the hundreds of coccinellids collected on *Helianthus* species between 2012 and 2025, none were observed feeding on aphids or pollen on the surfaces of the flowers. In most cases, lady beetles were found on or among the bracts on the backs of the inflorescences, on leaf petioles, or on the leaves. Observed lady beetles appeared to be directly damaging leaves and feeding on plant sap. (Figure 2 and Figure 3). In many cases, the lady beetles were observed chewing into the bracts, petioles, or leaves, appearing to feed on plant tissues, perhaps extrafloral nectaries [46,47].

Lady beetle apparent feeding on vegetation was observed most frequently in late summer, and there was a noteworthy shift in plant association over the course of the season from spring to fall. For *C. novemnotata*, based on month of capture, there was a strong association with *Helianthus* species from July to September, with over 90% of the specimens collected on *Helianthus* species during these three months (Table 3). In addition to the use of sunflower sap, it appears likely that sap-feeding occurs on several other plant species in Nebraska from July until September. We have observed apparent sap or plant exudation feeding by lady beetles on *Helianthus annuus*, *Helianthus petiolaris*, *Helianthus nuttallii*, *Cirsium canescens* Nutt., *Brickellia eupatorioides* (L.) Shinners, *Alcea rosea* Linnaeus, *Paeonia lactiflora* Pallas, and *Mentzelia nuda* (Pursh) Torr. & A. Gray.

Focused searches conducted with broadly similar effort revealed substantial differences in the number of adult coccinellids detected in 2024 and 2025. Many species were frequently encountered in 2024, particularly on sunflower, but substantially fewer individuals were observed or collected in 2025. For example, 88 *Coccinella novemnotata* were collected, and additional individuals were observed during 2024, whereas two individuals were observed and collected during 2025. Similarly, 124 *Hippodamia glacialis* were collected, and additional individuals were observed during 2024, whereas none were detected during 2025. These results demonstrate substantial interannual variation in detections during the two survey years. Because the surveys were not designed to estimate population density or detection probability, the differences should not be interpreted as quantified population declines between 2024 and 2025.

## 4. Discussion

Predaceous coccinellids provide an important ecosystem service by suppressing herbivorous insects, including aphids (Hemiptera: Aphididae), scale insects (Hemiptera: Coccoidea), whiteflies (Hemiptera: Aleyrodidae), psyllids (Hemiptera: Psylloidea), mites (Acari), and the eggs and larvae of beetles (Coleoptera) and moths (Lepidoptera) [48,49]. Both larvae and adults are voracious predators capable of consuming hundreds of prey items during development, substantially reducing pest populations before economic thresholds are reached [49,50]. Their effectiveness has led to widespread recognition of lady beetles as foundational components of integrated pest management (IPM) programs in agricultural systems worldwide. Several species have also been intentionally introduced as classical biological control agents, with notable successes against scale insects and aphids in orchards, citrus groves, and greenhouse production systems [51]. However, the ecological value of coccinellid collections extends beyond their role in biological control. Museum specimens and associated label data can document historical occurrence, phenology, geographic distributions, habitat associations, and changes in community composition [42,44]. Such records are particularly valuable in regions such as the Great Plains, where standardized long-term surveys are comparatively uncommon.

Beyond their direct contribution to pest suppression, lady beetles serve as indicators of habitat quality and ecological integrity. Because species differ substantially in habitat specialization, prey preferences, dispersal ability, and sensitivity to disturbance, changes in coccinellid communities often reflect broader environmental changes occurring within terrestrial ecosystems [6]. Native assemblages generally contain both habitat specialists and generalist predators whose coexistence contributes to ecological resilience through complementary resource use. Consequently, shifts in species composition may alter trophic interactions, predator-prey dynamics, and the stability of biological control services.

The ecological importance of Coccinellidae has stimulated extensive research into their taxonomy, phylogeny, behavior, and conservation. Molecular phylogenetic analyses have substantially revised the classification of the family during the past two decades, recognizing previously unappreciated evolutionary relationships and clarifying the placement of numerous tribes and genera [19]. Concurrently, improved taxonomic treatments and regional identification guides have facilitated biodiversity surveys across North America [45,52,53,54]. Despite these advances, significant geographic variation in sampling intensity continues to limit understanding of species distributions, particularly within the Great Plains and portions of the south-central United States.

Our study inventoried lady beetles of Nebraska that are held in the CSC Insect Collection and supplemented the collection records with additional natural history observations. More than 2500 specimens were inventoried in the collection, and it comprised recently sampled specimens of lady beetles whose relative abundance has declined considerably in the last few decades across North America [26,27,31,32]. In addition, natural history observations were made in 2025 of *A. bipunctata*, *C. novemnotata*, and in 2024 of *C. transversoguttata richardsoni*. Thus, these records confirm the importance of natural history observations and the use of natural history collections in documenting historical and recent trends in lady beetle biodiversity [23,24,42,43].

Collection information was available for 1882 Nebraska specimens, and approximately two-thirds of these specimens were obtained by hand collection. These proportions describe how specimens represented in the CSC collection were obtained, but care in interpreting the results must be exercised, as the results do constitute a comparison of sampling efficiency among methods. In our study, collection method and targeted plant searches strongly influenced which species were represented in the CSC dataset of lady beetles in western Nebraska. Sampling effort was not standardized across all collection methods. In particular, blacklight sampling was concentrated at a single location, whereas hand collections and sweep samples were obtained across broader areas and at different time periods. Museum specimens also accumulated under collection protocols that changed through time. Consequently, the percentage of specimens obtained by a particular method (Table 3) should not be interpreted as evidence that the method is inherently more effective than another method or that species represented by large numbers (Table 4) were necessarily more abundant in the landscape.

Despite these limits, the data reveal potentially useful method-specific detection patterns. More than half of the CSC specimens of *Adalia bipunctata*, *Harmonia axyridis*, *Hippodamia tredecimpunctata tibialis*, *Myzia interrupta*, and *Olla v-nigrum* originated from a single continuously operated blacklight. Light trapping has historically been used less frequently than visual searching, sweeping, or beat sheets in surveys for coccinellids [43,55], though previous studies have documented attraction of *H. axyridis* and *H. redecimpunctata tibialis* to lights [56,57,58]. In fact, *Harmonia axyridis* often dominates blacklight captures of coccinellids [56,57,58], and this has been attributed to its relatively higher scotophase activity [58]. Thus, the bias of blacklight trapping may be useful as a potential control strategy for *H. axyridis* [59] or for selective sampling of night-active coccinellids. The frequent captures of *A. bipunctata*, *M. interrupta*, and *O. v-nigrum* in our study indicate that nocturnal flight activity may be more common in these three species than generally documented. The CSC records therefore suggest that blacklight trapping may complement conventional daytime methods for detecting some coccinellid species, but the present data cannot be used to quantify the relative efficiency of light traps because trap effort was not replicated among sites or standardized against the other collection methods.

Similarly, the relatively high number of specimens of *Coccinella transversoguttata richardsoni* from yellow pan traps suggests that passive trapping methods may improve detection of some taxa that are otherwise infrequently encountered by conventional sampling. These findings indicate that comprehensive biodiversity surveys, even for popular well-studied groups, should incorporate multiple complementary and standardized sampling techniques to reduce method-specific detection bias and improve estimates of species richness, occupancy, and, where sampling effort permits, relative abundance. However, experimental comparisons using equal sampling effort are needed before concluding that pan traps increase detection probability relative to other methods.

An additional source of bias in our data is body size and detectability. The present study intentionally emphasizes the larger and more readily identifiable Coccinellidae, and much of the recent material was obtained by visual hand collection. Visual searches are inherently more likely to detect conspicuous, larger-bodied beetles than small or cryptic species. Consequently, groups of small Coccinellidae, including members of Microweiseini, Scymnini, Diomini, and Hyperaspini [45], are almost certainly underrepresented in the CSC dataset. The 35 identified species documented here should therefore not be interpreted as a complete inventory of western Nebraska Coccinellidae. Future regional inventories should combine standardized visual searches, sweep and beat sampling, passive traps, light traps, and methods specifically targeting small or cryptic taxa.

Plant-association records and recent field observations identified native sunflowers (*Helianthus* spp.) as frequently used substrates by several coccinellid species during the latter portion of the active season. Numerous species, including *Coccinella novemnotata*, *C. monticola*, and *Hippodamia convergens*, were frequently collected from sunflowers during late summer. These observations were consistent with previous reports from western Nebraska of various adult coccinellids associated with roadside vegetation, including *Helianthus* spp. [42,43]. Our field observations documented apparent feeding on leaves, petioles, and bracts rather than on aphids or pollen by coccinellids. Although coccinellids are widely recognized as predaceous, supplemental feeding on pollen, nectar, fungi, and plant tissues has been reported in several taxa and may provide water or carbohydrates when prey become scarce [46].

These observations are consistent with supplemental phytophagy or consumption of plant exudates, but the nutritional source was not experimentally determined. Thus, the observations should be interpreted as evidence of plant-associated feeding behavior rather than definitive evidence that plant tissue or sap constituted the primary food source. Experimental observations of feeding, gut-content analysis, or stable-isotope approaches could help distinguish direct herbivory from consumption of exudates, pollen, fungi, or small prey not detected during visual observations.

Concentrations of beetles on particular plants also complicate interpretation of collection counts. Coccinellids may aggregate in response to concentrated prey, pollen or nectar resources, plant exudates, reproductive behavior, or chemical and visual cues. Individuals may also remain on vegetation after local aphid populations have declined. Consequently, large numbers collected from individual sunflower patches could reflect temporary aggregation rather than high landscape-scale population density. This effect is especially relevant when targeted visual searches focus on plants already known to harbor beetles. Collection records from such aggregations are biologically informative because they identify potentially important resources and behaviors, but they should not be treated as unbiased estimates of regional abundance.

The repeated observations of apparent sap or plant tissue feeding across multiple native and introduced plant species suggest that this behavior may be more widespread among Great Plains coccinellids than previously recognized. The difference between the 2024 and 2025 collections illustrates this issue. In 2024, 88 *C. novemnotata* were collected, and many additional individuals were observed, whereas only two were observed and collected in 2025. Likewise, 124 *H. glacialis* were collected in 2024 and none in 2025. Because search effort in the two years was broadly similar but not part of a fully replicated standardized monitoring design, these observations demonstrate strong interannual variation in detections rather than a quantified population decline between years. The mechanisms responsible for this variation are unknown. Differences in prey abundance, dispersal, reproductive success, overwintering survival, weather, and the formation or location of plant-associated aggregations could all contribute and warrant experimental investigation. Because several native coccinellid species were repeatedly recorded from native prairie forbs, particularly *Helianthus*, future research should test whether maintaining diverse native forb communities provides important prey, plant-derived foods, refugia, or other resources for these species.

Aphid availability deserves additional consideration because aphidophagous coccinellids frequently track spatial and temporal variation in prey populations. Declining aphid availability can stimulate dispersal or increased use of alternative foods, including pollen, nectar, and plant-derived resources. The unusually large aggregations observed on sunflower in 2024 could have resulted partly from changes in prey availability, but aphid abundance was not quantified, and this hypothesis cannot be tested with the present data. Future surveys that simultaneously quantify coccinellids, aphids, floral resources, and plant condition would provide a stronger basis for interpreting these associations.

Multiple anthropogenic stressors have been implicated in insect declines, with habitat loss and landscape modification consistently identified as dominant drivers [11,60,61]. Conversion of natural ecosystems to agriculture, urban development, and infrastructure fragments previously continuous habitats, reduces resource availability, and isolates populations. Intensive agricultural systems frequently replace structurally diverse landscapes with extensive monocultures that provide limited floral resources, nesting substrates, and overwintering habitat for insects [62,63]. Simplification of agricultural landscapes also disrupts trophic interactions by reducing predator diversity and altering host–parasitoid relationships, thereby influencing the stability of ecological communities [63]. In our study, a substantial number and diversity of coccinellids were sampled from unmanaged roadsides, whereas many roadsides in rural areas are managed with herbicides and mowing [64]. The effects of roadside management have been frequently considered for pollinators [64]. Based on our findings, a lack of roadside management may benefit lady beetles by providing plant resources, although our study did not explicitly test the effects of roadside management on occupancy of coccinellids in habitats adjacent to roads.

Changes in agricultural management have compounded the effects of habitat loss. Increased mechanization, removal of vegetative borders, widespread herbicide and insecticide application, reduced crop diversity, and frequent disturbance collectively decrease habitat heterogeneity across agricultural regions [64]. Although modern agricultural practices have substantially increased food production, they have often done so at the expense of biodiversity associated with semi-natural habitats embedded within production landscapes. Landscape-scale studies consistently demonstrate that insect richness and abundance increase with greater proportions of natural vegetation surrounding agricultural fields, emphasizing the importance of habitat connectivity for maintaining insect communities [63,65].

The role of pesticides in insect declines has received considerable scientific attention. Broad-spectrum insecticides may directly reduce survival of target and non-target species while indirectly affecting insect populations through altered prey availability, behavioral modification, or disruption of ecological interactions [66]. Neonicotinoid insecticides have been particularly investigated because of their systemic properties, water solubility, and documented effects on pollinators [67,68]. Laboratory experiments demonstrate sublethal impacts on navigation, reproduction, immune function, and behavior in numerous insect taxa, although translating laboratory findings to landscape-scale population effects remains challenging because of interacting environmental variables [69]. Consequently, current evidence suggests that pesticide exposure acts with habitat loss and other stressors rather than functioning as an isolated cause of decline [14,70].

Climate change represents another increasingly important driver of insect population dynamics. Rising temperatures influence development rates, voltinism, diapause, phenology, and geographic distributions while simultaneously altering interactions among predators, prey, competitors, and host plants [71,72]. Numerous temperate insects have expanded northward or moved to higher elevations in response to warming climates, whereas species with narrow climatic tolerances or restricted geographic ranges have experienced population declines [72]. Here, records show that *C. maculata* has expanded its westward distribution throughout western Nebraska since the 1970s [73]. Highly variable weather events, including prolonged droughts, heat waves, flooding, and late-season freezes, further contribute to insect mortality and reproductive failure.

It is especially noteworthy that many coccinellid species in western Nebraska were frequently encountered during focused surveys in 2024 (Table 4), especially on sunflower. Many of these same species were detected much less frequently during focused surveys in 2025, despite sunflower populations appearing relatively unchanged. Although 88 *C. novemnotata* were collected, with many more seen in 2024, only two individuals were seen and collected in 2025. A total of 124 *H. glacialis* were collected, and many more were seen in 2024, but none were found in 2025. Although the reasons for differences between years are not known, perhaps exploitation of sunflower sap by many adult coccinellid species in 2024 was driven by food limitation, as proposed by Michaud and Qureshi [74]. Climate change interacts with habitat fragmentation and land-use changes to produce cumulative ecological effects and can affect the prey base and higher trophic levels [11,14]. Although native sunflower species attracted large numbers of many coccinellid species and offered a very effective way to sample the adults, testing of other factors, including prey availability, is required in future studies.

Additional drivers contributing to insect declines include invasive species, pollution, altered fire regimes, artificial light at night (ALAN), and emerging pathogens [75,76]. Artificial lighting has received increasing attention because it disrupts nocturnal insect navigation, mating behavior, predator avoidance, and circadian rhythms while altering predator–prey interactions [76]. Species collected at blacklight are presumably dispersing at night and more likely to be affected by ALAN, potentially explaining declines of some species in urban areas. Likewise, invasive plants and arthropods can restructure native communities through competition, predation, and modification of habitat characteristics. These interacting stressors create cumulative pressures that frequently exceed the effects of any single environmental factor [77].

The geographical range expansion of *H. axyridis* has attracted particular attention because its establishment has coincided with declines of several native lady beetles in eastern North America. Multiple mechanisms have been proposed to explain these associations, including exploitative competition for shared prey resources, intraguild predation, pathogen transmission, and asymmetric reproductive or behavioral interactions [78,79]. Laboratory experiments demonstrate that *H. axyridis* frequently consumes eggs and larvae *H. axyridis* frequently consumes eggs and larvae of native coccinellids while exhibiting greater synthesis of defensive alkaloids and more resistance to some fungal pathogens than many native competitors pathogens than many native competitors [78]. The first record of *H. axyridis* in western Nebraska is 2005, and there is currently only a total of 107 specimens, suggesting that its population has not increased as dramatically in the area as it has in other parts of the United States. In contrast to *H. axyridis*, the introduced *C. septempunctata* is one of the two most frequently collected coccinellid species in western Nebraska, and only slightly less frequently than *H. convergens*. Changing distributions of lady beetle species driven by weather patterns may contribute to losses or increases in the community. Rather than introduced species alone causing documented declines, evidence increasingly supports a multifactorial hypothesis in which biological invasions interact with habitat alteration, changing agricultural practices, climate, and landscape simplification to restructure native communities [26,32,80].

Despite documented changes in insect biomass and communities, responses have not been uniform among taxa. Several native species remain common or have expanded regionally, suggesting that life-history traits, habitat specialization, and landscape context influence vulnerability to decline. For example, generalist predators capable of exploiting disturbed habitats may be more resilient than habitat specialists dependent upon prairie remnants, forest edges, or specific perennial vegetation. Some native North American coccinellid species have experienced well-documented declines over recent decades [17], making regional inventories particularly valuable for evaluating contemporary patterns of species occurrence and community composition [81]. As a consequence, regional surveys remain essential for identifying species-specific responses necessary to evaluate whether community changes observed elsewhere [8] are occurring across under-sampled landscapes.

Long-term monitoring has further demonstrated that coccinellid assemblages are dynamic and can respond rapidly to environmental change [81]. Because many species exhibit strong seasonal movements among agricultural fields, grasslands, forests, and urban habitats, comprehensive inventories require repeated sampling across habitat types and throughout the growing season. Repeated surveys conducted with standardized effort would provide baseline data necessary for detecting future changes in species richness, relative abundance, phenology, occupancy, and geographic distribution. As insect conservation increasingly emphasizes standardized biodiversity monitoring, lady beetles represent an excellent focal group because their taxonomy is comparatively well resolved, historical records are extensive, and their ecological importance is well established.

## 5. Conclusions

Despite increasing evidence of insect declines, substantial knowledge gaps remain regarding temporal and spatial patterns of biodiversity change. Existing long-term datasets are heavily concentrated in Europe and selected regions of North America, while many portions of the world remain poorly sampled [11,14]. Even within the United States, monitoring efforts are geographically uneven, with relatively few standardized inventories conducted across the Great Plains, Interior Highlands, and portions of the south-central states. Taxonomic biases further complicate interpretation because charismatic groups such as butterflies and bees have received considerably greater attention than predatory beetles, parasitoids, flies, or many aquatic insects. Consequently, broad conclusions regarding insect decline often rely upon a limited subset of insect diversity.

Long-term biodiversity inventories provide the foundation for understanding these patterns. Repeated surveys establish baseline estimates of species richness, abundance, community composition, and geographic distribution against which future ecological changes can be evaluated [82]. Such inventories also facilitate detection of invasive species, document range expansions associated with climate change, identify species of conservation concern, and provide voucher specimens for future taxonomic revision. Museum collections derived from historical inventories are particularly valuable because they document species occurrences and distributions across decades or centuries. Where historical sampling effort is sufficiently documented and standardized, such collections may also contribute to quantitative analyses of temporal changes; where effort is heterogeneous, as in the present dataset, their strongest application is reconstruction of occurrence, phenology, distribution, and natural-history patterns [83].

Beyond documenting biodiversity, inventories contribute directly to ecological theory and conservation planning [44]. Knowledge of species distributions informs habitat management, restoration prioritization, protected area design, and biological control programs. Regional inventories are especially valuable in under-sampled areas where species distributions remain incompletely documented, and historical baseline data are lacking. As environmental conditions continue to change, repeated standardized surveys become increasingly important for distinguishing natural fluctuations from persistent population declines and for identifying taxa requiring conservation attention. Late-season standardized surveys for lady beetles, particularly species that have undergone documented declines elsewhere such as *C. novemnotata*, should include systematic searches of *Helianthus* and other native forbs to determine whether plant-use patterns documented in western Nebraska occur elsewhere [42,43].

## Figures and Tables

**Table 1:** Specimens of large Coccinellidae in the insect collection at Chadron State College (CSC) in western Nebraska. Percentage values indicate the proportion of Nebraska coccinellid specimens represented by each species in the collection and do not represent standardized estimates of relative field abundance.

Species	Nebraska Specimens in CSC Collection	Most Recent Collection Date	Percentage of Nebraska CSC Specimens
*Adalia bipunctata*	79	2025	3.59%
*Anatis labiculata*	23	2024	1.05%
*Anatis lecontei*	1	1960	0.05%
*Anatis mali*	21	2024	0.95%
*Brachiacantha albifrons*	11	2024	0.50%
*Brachiacantha tau*	50	2020	2.27%
*Brachiacantha uteella*	6	2024	0.27%
*Calvia quatuordecimguttata*	16	2024	0.73%
*Chilocorus stigma*	1	1975	0.05%
*Coccinella difficilis*	1	2024	0.05%
*Coccinella monticola*	6	2024	0.27%
*Coccinella novemnotata*	122	2025	5.55%
*Coccinella prolongata*	1	1946	0.05%
*Coccinella septempunctata*	551	2025	25.05%
*Coccinella transversoguttata richardsoni*	34	2024	1.55%
*Coleomegilla maculata*	26	2025	1.18%
*Cycloneda munda*	2	2022	0.09%
*Epilachna varivestis*	8	2003	0.36%
*Exochomus aethiops*	3	2024	0.14%
*Harmonia axyridis*	107	2025	4.86%
*Hippodamia apicalis/expurgata*	5	2019	0.23%
*Hippodamia caseyi*	1	1965	0.05%
*Hippodamia convergens*	606	2025	27.55%
*Hippodamia expurgata*	10	2024	0.45%
*Hippodamia glacialis*	154	2024	7.00%
*Hippodamia quindecimmaculata* (likely)	3	2025	0.14%
*Hippodamia quinquesignata*	1	1974	0.05%
*Hippodamia parenthesis*	229	2025	10.41%
*Hippodamia tredecimpunctata tibialis*	75	2024	3.41%
*Mulsantina picta*	11	2025	0.50%
*Myzia interrupta*	5	1991	0.23%
*Olla v-nigrum*	14	2013	0.64%
*Psyllobora vigintimaculata*	5	2015	0.23%
*Scymnus brullei*	10	2012	0.45%
*Scymnus caudalis*	2	2012	0.09%

**Table 2:** Representation of selected coccinellid species in the Chadron State College Insect Collection by recorded collection method and associated plant. Percentages indicate the proportion of archived specimens of each species associated with a particular collection method or plant. Sampling effort was not standardized among methods, sites, or time periods; therefore, percentages should not be interpreted as estimates of sampling efficiency, detection probability, habitat preference, or relative abundance. Totals within rows may be <100% because some specimens lacked information on collection method or associated plant.

		Percent by Collection Method/Plant Species						
Species	Total (N)	Light	Yellow Pan Trap	Alfalfa (Sweep)	*Helianthus* sp.	*Ulmus* (Sweep)	*Persicaria* (Sweep)	*Ribes*
*Adalia bipunctata*	79	72	0	0	0	0	0	5
*Anatis labiculata*	23	17	0	0	0	0	0	0
*Analis mali*	21	19	0	0	24	0	0	0
*Coccinella monticola*	6	0	0	0	100	0	0	0
*Coccinella novemnotata*	122	0	1	0	75	0	0	0
*Coccinella septempunctata*	551	1	5	13	30	0	0	1
*Coccinella transversoguttata*	34	0	26	0	21	0	0	0
*Coleomegilla maculata*	26	0	5	0	0	0	50	0
*Harmonia axyridis*	107	55	0	11	12	0	0	2
*Hippodamia convergens*	606	6	7	3	31	0	1	1
*Hippodamia expurgata*	10	0	0	0	90	0	0	0
*Hippodamia quindecimmaculata* (likely)	3	0	0	0	67	0	0	0
*Hippodamia parenthesis*	229	4	0	27	16	0	0	0
*Hippodamia tredecimpunctata*	75	88	0	0	0	0	4	0
*Mulsantina picta*	11	27	0	0	18	0	0	0
*Myziua interrupta*	5	80	0	0	0	0	0	0
*Olla v-nigrum*	14	79	0	0	0	0	0	0
*Scmnus brullei*	10	0	0	0	0	100	0	0
*Scmnus caudalis*	2	0	0	0	0	50	0	0

**Table 3:** Collection-label records for *Coccinella novemnotata* by month, collection method, and recorded plant association in western Nebraska, USA, 1990–2025. Values represent specimen records rather than standardized estimates of monthly abundance or plant preference. “N/A” indicates that collection method or plant-association information was unavailable.

Month	Total	N/A	Hand	USDA Grassshopper Sweeps	On *Artemisia tridentata*	On *Brickellia eupatoroides*	On *Helianthus* sp.
May	4	1	2	2	1	0	0
June	12	0	5	1	0	0	7
July	46	0	1	0	0	1	42
August	26	1	3	0	0	0	21
September	35	0	0	0	0	0	36

**Table 4:** Numbers of adult lady beetles collected during focused surveys near Chadron, Nebraska, USA, in 2024 and 2025. Search effort was broadly similar between years (approximately 40–50 h annually across 15–25 survey days), but the surveys were not designed as a replicated population-monitoring study. Values therefore represent annual collection/detection totals and should not be interpreted as estimates of population density or percentage population change.

	Number Collected	
Species	2024	2025
*Adalia bipunctata*	4	2
*Anatis labiculata*	6	0
*Aanatis mali*	6	0
*Calvia quatuordecimguttata*	2	0
*Coccinella monticola*	6	0
*Coccinella novemnotata*	88	2
*Coccinella septempunctata*	168	59
*Coccinella transversoguttata richardsoni*	8	0
*Coleomegilla maculata*	11	1
*Exochomus aethiops*	3	0
*Harmonia axyridis*	16	5
*Hippodamia convergens*	207	52
*Hippodamia expurgata*	10	0
*Hippodamia glacialis*	124	0
*Hippodamia parenthesis*	106	3
*Hippodamia tredecimpunctata tibialas*	1	0
*Mulsantina picta*	2	2

## Data Availability

All data used in this paper are presented here-in. Specimens are deposited in the Chadron State College Insect museum and are available upon request to the corresponding author. The coccinellid collection at CSC has been fully inventoried and databased.

## Appendix A. Table of All Species and Records

Below, we briefly discuss the Nebraska Coccinellid species contained in the CSC collection and provide a brief synopsis of what can be discerned about each from the data obtained as well as recent observations. Each species is discussed separately, and an associated data table is provided, as well as photographs for some species.

*Adalia bipunctata* (Linnaeus): A total of 79 specimens are contained in the CSC collection. Of these, over 70% were collected in the 1970s and 1980s, mostly at lights in or near Chadron, Nebraska. A few series of more than three specimens collected in a single night suggest this species was likely common in the area at the time. A total of 14 specimens were collected between 2013 and 2025, with six collected in the past two years. In 2024, the first author collected four specimens and observed at least two more on a single currant (*Ribes* sp.) shrub in a yard in Chadron. All were remarkably similar in phenotype.

*Anatis labiculata* (Say): A total of 23 Nebraska specimens are contained in the CSC collection (Table 1). This species appears to be mostly associated with riverine woodlands in western Nebraska, and this species was found in good numbers among grasses in the woodland understory along the Campground Trail at Chadron State Park in 2024. All of these 2024 specimens were very pale in color, suggesting they were teneral.

*Anatis lecontei* Casey: The CSC collection contains a single Nebraska specimen collected in Dawes County in 1960. This species is unmistakable due to its large size and unique appearance.

*Anatis mali* (Say): A total of 21 Nebraska specimens are contained in the CSC collection. The most recent collections in 2024 included one specimen collected on *Helianthus annuus* or *H. petiolaris* in Sioux County and four specimens collected on *Helianthus nuttallii* in wetland habitat in Cherry County.

*Brachiacantha albifrons* (Say): Eleven Nebraska specimens are contained in the CSC collection. Only one specimen has been collected more recently than 1989. Although two Nebraska specimens were collected and photographed from Dawes County in 2024, one escaped while being transferred to a vial of alcohol. The two specimens from 2024 were collected in a sweep net sample from among roadside alfalfa.

*Brachiacantha tau* Le Conte: Fifty Nebraska specimens are contained in the CSC collection. Although most specimens were collection were collected prior to 1991, 16 specimens were collected in 2019, nearly all on *Artemisia tridentata* at Toadstool Geologic Park in Sioux County.

*Brachiacantha uteella* Casey: The CSC collection contains only 6 Nebraska specimens, all of which were collected in Dawes County. Of these, 5 were collected in 2024 by sweep net samples among smartweed (*Persicaria* sp.).

*Calvia quatuordecimguttata* (Linnaeus): Sixteen Nebraska specimens are contained in the CSC collection. The most specimens collected in a single year were in 2012, with 5 specimens collected. Although one was collected on *Helianthus*, most specimens in 2012 and 2024 were collected from tree leaves such as those of *Ulmus americana*.

*Chilocorus stigma* (Say): The CSC collection contains a single Nebraska specimen collected in Dawes County in 1975.

*Coccinella difficilis* Crotch: The CSC collection contains a single Nebraska specimen that was collected in Sioux County in 2024.

*Coccinella monticola* Casey: Six Nebraska specimens are contained in the CSC collection. All of these specimens were collected in 2024 in Sioux and Dawes Counties, and all were collected on either *Helianthus annuus* or *H. petiolaris*.

*Coccinella novemnotata* Herbst: A total of 122 Nebraska specimens are contained in the CSC collection. Of this total, 90 of the specimens were collected in 2024 (88) and 2025 (2), primarily in Dawes and Sioux Counties. The first author observed many additional individuals in 2024 in both Dawes and Sioux Counties. Recent records confirm the continued occurrence of *C. novemnotata* in Dawes and Sioux Counties. Within recent collections and observations, substantially fewer *C. novemnotata* were encountered than *C. septempunctata* (ratio of about 50 to 1) or *H. convergens* (ratio of about 200 to 1). Because survey effort was not standardized across all locations and years, these observations should not be interpreted as estimates of relative population density.

*Coccinella prolongata* Crotch: The CSC collection contains a single Nebraska specimen collected in Sioux County in 1946. Two specimens were photographed but not collected in Sioux County in 2018 and again in 2021.

*Coccinella septempunctata* Linnaeus: With 551 Nebraska specimens, *C. septempunctata* is one of the two species most frequently represented in the CSC Nebraska collection, exceeded only by *H. convergens*. Its frequent occurrence in recent observations also confirms that the introduced species is well established in western Nebraska.

*Coccinella transversoguttata richardsoni* Brown: A total of 34 Nebraska specimens are contained in the CSC collection. The records are broadly spread between 1965 and 2024, with no more than eight specimens from any year. The most specimens from a single year are from 2024, when 8 specimens were collected.

*Coleomegilla maculata lengi* Timberlake: A total of 26 Nebraska specimens are contained in the CSC collection. As with the previous species, the records are spread broadly over time, with only 1–3 specimens collected in a single year between 1974 and 2015. However, one specimen was collected in Cherry County, and 10 specimens were collected in Dawes County in 2024, with the 10 Dawes County specimens collected by sweep net samples among smartweed (*Persicaria* sp.). Records show that this species has expanded its westward distribution throughout western Nebraska since the 1970s [73].

*Cycloneda munda* (Say): Two Nebraska specimens are contained in the CSC collection. Both specimens were collected in central Nebraska. This is generally an eastern species and is not expected to occur in the Chadron area.

*Epilachna varivestis* Mulsant: Eight Nebraska specimens are contained in the CSC collection. All were collected between 1965 and 2003.

*Exochomus aethiops* (Bland): Three Nebraska specimens are contained in the CSC collection. All were collected in 2024 in Sioux County on either *Helianthus annuus* or *H. petiolaris*.

*Harmonia axyridis* (Pallas): Although *H. axyridis* is widely established and frequently encountered in many portions of eastern North America, only 107 Nebraska specimens are represented in the CSC collection, more than half of which were obtained at lights in Dawes County. CSC records and recent observations suggest that the species is less prominently represented in the Nebraska Panhandle than *C. septempunctata* or *H. convergens*. Many western Nebraska records are associated with riverine woodland settings, although the available records are insufficient to demonstrate habitat preference.

*Hippodamia apicalis/expurgata*: Five Nebraska specimens are contained in the CSC collection. All were collected between 1940 and 2019, and all are most likely *Hippodamia expurgata*.

*Hippodamia caseyi* Johnson: The CSC collection contains a single Nebraska specimen collected in Scotts Bluff County in 1965.

*Hippodamia convergens* (Guerin): With 606 Nebraska specimens, *H. convergens* is the species most frequently represented in the CSC Nebraska collection. It was also frequently encountered during recent field observations. Because historical sampling effort was heterogeneous, its numerical representation relative to *C. septempunctata* should not be interpreted as an estimate of their relative population densities.

*Hippodamia expurgata* Casey: Ten Nebraska specimens are contained in the CSC collection. All were collected in 2024, mostly on either *Helianthus annuus* or *H. petiolaris*.

*Hippodamia glacialis* (Fabricius): A total of 154 Nebraska specimens are contained in the CSC collection. Over 90% of the specimens were collected in 2012 and 2024 and mostly on either *Helianthus annuus* or *H. petiolaris*. Recent observations indicate substantial interannual variation in detection of this species in western Nebraska. More than 120 individuals were collected, and additional individuals were observed in 2024, whereas none were detected during comparable focused searches in 2025. This difference demonstrates strong interannual variation in detections. However, two survey years cannot establish the magnitude or direction of a population trend.

*Hippodamia quindecimmaculata* Mulsant (unconfirmed): Three Nebraska specimens are contained in the CSC collection. One was collected in north-central Nebraska (Holt County) in 2018, and its identification remains unclear. A Dawes County specimen from 2024 appears to very likely be this species, while a Dawes County specimen from 2025 appears to more likely be *H. convergens*.

*Hippodamia quinquesignata* (Kirby): The CSC collection contains a single Nebraska specimen collected in Scotts Bluff County in 1974.

*Hippodamia parenthesis* (Say): The 229 Nebraska specimens in the CSC collection include records from multiple locations, with many records associated with grassland habitats. Relatively few available specimens were recorded from *Helianthus* spp. compared with several other *Hippodamia* species examined in this study. These collection records suggest differences in plant use or detectability that warrant testing with standardized habitat and plant surveys. *Hippodamia tredecempunctata tibialis* (Say): A total of 75 Nebraska specimens are contained in the CSC collection. Although three were collected by sweep net among smartweed (*Persicaria* sp.), most were collected at blacklights.

*Mulsantina picta* (Randall): Eleven Nebraska specimens are contained in the CSC collection. The specimens were collected primarily by hand, at lights, or on either *Helianthus annuus* or *H. petiolaris*.

*Myzia interrupta* (Casey): Five Nebraska specimens are contained in the CSC collection. Most were collected at lights, with the most recent collection in 1991.

*Olla v-nigrum* (Mulsant): Fourteen Nebraska specimens are contained in the CSC collection. Most were collected at lights, with the most recent collection in 2013.

*Psyllobora vigintimaculata* (Say): Five Nebraska specimens are contained in the CSC collection. All were collected between 1976 and 2015, and the collection methods were not recorded.

*Scymnus brullei* Mulsant: Ten Nebraska specimens are contained in the CSC collection. All were identified by dissection of the genitalia by the second author. All were collected by sweep net among leaves of elm (most likely American elm).

*Scymnus caudalis* LeConte: Two Nebraska specimens are contained in the CSC collection. Both were identified by dissection of the genitalia by the second author. One was collected in 1979, and the other was collected by sweep net among leaves of elm (most likely American elm).
